# Identification of a Gene Signature Predicting (Nano)Particle-Induced Adverse Lung Outcome in Rats

**DOI:** 10.3390/ijms241310890

**Published:** 2023-06-29

**Authors:** Sarah Amandine Valentino, Carole Seidel, Mylène Lorcin, Sylvie Sébillaud, Henrik Wolff, Stéphane Grossmann, Stéphane Viton, Hervé Nunge, Laura Aliisa Saarimäki, Dario Greco, Frédéric Cosnier, Laurent Gaté

**Affiliations:** 1 French Research and Safety Institute for the Prevention of Occupational Accidents and Diseases (INRS), Toxicology and Biomonitoring Division, 1 Rue du Morvan, F-54519 Vandœuvre-lès-Nancy, France; 2 Finnish Institute of Occupational Health, FI-00251 Helsinki, Finland; henrik.wolff@ttl.fi; 3 Finnish Hub for Development and Validation of Integrated Approaches (FHAIVE), Faculty of Medicine and Health Technology, Tampere University, FI-33520 Tampere, Finland; 4 Division of Pharmaceutical Biosciences, Faculty of Pharmacy, University of Helsinki, FI-00100 Helsinki, Finland

**Keywords:** gene signature, phospholipids, inhalation, nanoparticles, crystalline silica, titanium dioxide, carbon black, multi-walled carbon nanotube

## Abstract

Nanoparticles are extensively used in industrial products or as food additives. However, despite their contribution to improving our quality of life, concerns have been raised regarding their potential impact on occupational and public health. To speed up research assessing nanoparticle-related hazards, this study was undertaken to identify early markers of harmful effects on the lungs. Female Sprague Dawley rats were either exposed to crystalline silica DQ-12 with instillation, or to titanium dioxide P25, carbon black Printex-90, or multi-walled carbon nanotube Mitsui-7 with nose-only inhalation. Tissues were collected at three post-exposure time points to assess short- and long-term effects. All particles induced lung inflammation. Histopathological and biochemical analyses revealed phospholipid accumulation, lipoproteinosis, and interstitial thickening with collagen deposition after exposure to DQ-12. Exposure to the highest dose of Printex-90 and Mitsui-7, but not P25, induced some phospholipid accumulation. Comparable histopathological changes were observed following exposure to P25, Printex-90, and Mitsui-7. Comparison of overall gene expression profiles identified 15 potential early markers of adverse lung outcomes induced by spherical particles. With Mitsui-7, a distinct gene expression signature was observed, suggesting that carbon nanotubes trigger different toxicity mechanisms to spherical particles.

## 1. Introduction

Nanoparticles (NPs) are extensively used in the manufacturing of industrial products or as food additives [1,2,3]. However, while they may improve our quality of life, concerns have been raised regarding their potential impact on occupational and public health [4,5].

Like their micrometric counterparts, various NPs were originally considered to be materials with a low toxicity and low solubility. However, the larger surface area of NPs means that, for the same lung-deposited mass as micrometric particles, they exhibit a higher pulmonary toxicity despite an identical chemical composition. Carbon blacks (CBs) and titanium dioxides (TiO_2_) are considered low-toxicity and low-solubility NPs. TiO_2_ are widely used in everyday products as a white pigment, for example, in paints [6]. They are also used in cosmetics as UV filters [6]. The worldwide annual production of CBs is 10 million tons, and these particles are used in numerous industrial applications including the production of rubber, tires, paints, toners, and printing inks [7]. Other carbon-based NPs, including multi-walled carbon nanotubes (MWCNTs), are widely produced and used in the fields of electronics or sports, where their wide-ranging promising physical and chemical properties are harnessed [8]. During industrial use of any of these particles, especially when handling dry powders, workers may be exposed through inhalation [9,10,11], leading to pulmonary deposition that may have deleterious effects. These potential effects need to be assessed.

Anticipating the long-term adverse effects of NPs is crucial for the development of safer-by-design NPs in an ever-evolving world where economic and safety considerations go hand in hand. However, today, almost no data are available to predict the long-term toxic effects of these particles. In the past few years, high-throughput and high-content screening has been used to decipher the toxicity mechanisms of chemicals and identify biomarkers, predicting their toxicity and the related adverse outcomes (AO), such as chronic inflammation, lipoproteinosis, fibrosis, and cancer [12,13,14]. Exposure to crystalline silica, such as DQ-12, may lead to pulmonary fibrosis. This chemical has become the reference material for experimental inhalation toxicology [15,16,17,18]. In lung fibrosis induced by crystalline silica or bleomycin in rats, phospholipid concentrations in lung lavage fluid are reported to increase during the acute and chronic stages of the disease. The increase in phospholipids is considered to be involved in disease progression since phospholipid concentrations increase before pathological symptoms emerge [19,20].

With this study, we aimed to identify potential early biomarkers predictive of long-term adverse effects of NPs. To do so, we exposed rats to four benchmark materials either with subacute inhalation (CB (Printex-90, P90), TiO_2_ (P25), or MWCNT (Mitsui-7)) or with intratracheal instillation (crystalline silica (DQ-12)). Bronchoalveolar lavage (BAL) and lung tissue were collected. Inflammation, protein secretion, and phospholipid content were analyzed in BAL. Histopathology and gene expression were assessed in lung tissue. An analysis of overall gene expression profiles focused initially on identifying differentially expressed genes (DEGs) that could serve as predictive markers of long-term effects of spherical NPs (i.e., P90, P25, and DQ-12). This list was then compared to the list of DEGs detected following inhalation of Mitsui-7 (classified as a class 2B carcinogen by the International Agency for Research on Cancer).

## 2. Results

### 2.1. Pulmonary Exposure to Crystalline Silica, Titanium Dioxide, Carbon Black, and Carbon Nanotube-Induced Inflammation and Cytotoxicity

#### 2.1.1. Lung Biometry, Bronchoalveolar Lavage Cytology, and Biochemistry

**Figure 1:**
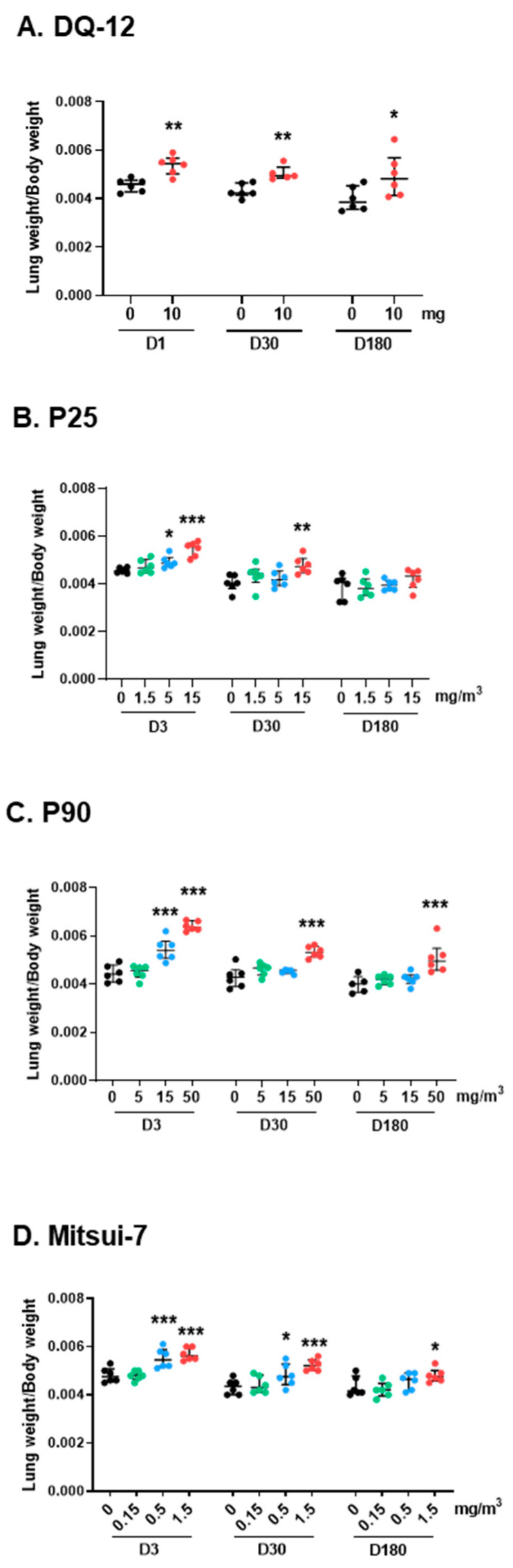
Lung weight following exposure to DQ-12, P25, P90, or Mitsui-7. Lungs and animals were weighed at the times indicated after the end of exposure to (**A**) 10 mg/rat of DQ-12; (**B**) 1.5, 5, or 15 mg/m^3^ of P25; (**C**) 5, 15, or 50 mg/m^3^ of P90; or (**D**) 0.15, 0.5, or 1.5 mg/m^3^ of Mitsui-7. The results are presented as a ratio of lung-to-body weight. Data are expressed as median [Q1; Q3]. * *p* < 0.05, ** *p* < 0.01, *** *p* < 0.001 significantly different from the control (*n* = 6).

Following the intratracheal instillation of crystalline silica DQ-12 (10 mg/rat), the ratio of lung-to-body weight was higher than that of control animals at the three post-exposure times (Figure 1A). The lung-to-body weight ratio of rats exposed with inhalation to 5 and 15 mg/m^3^ of P25 was also higher at 3 days (D3), and this increase was still observed at the highest dose at 30 days (D30, Figure 1B). The lung-to-body weight ratio of all rats exposed to P25 had returned to control levels at 180 days (D180). After the inhalation of 15 and 50 mg/m^3^ of P90, we also observed an increase in the lung-to-body weight ratio at 3 days (Figure 1C). This increase was still observed in the group exposed to the highest dose of P90 at D30 and D180. On D3 and D30 post exposure to Mitsui-7 (0.5 and 1.5 mg/m^3^), the lung-to-body weight ratio was higher than in control animals (Figure 1D). This increase persisted in the high-dose group at D180. Body weights and absolute lung weights are presented in Supplementary Table S1.

**Figure 2:**
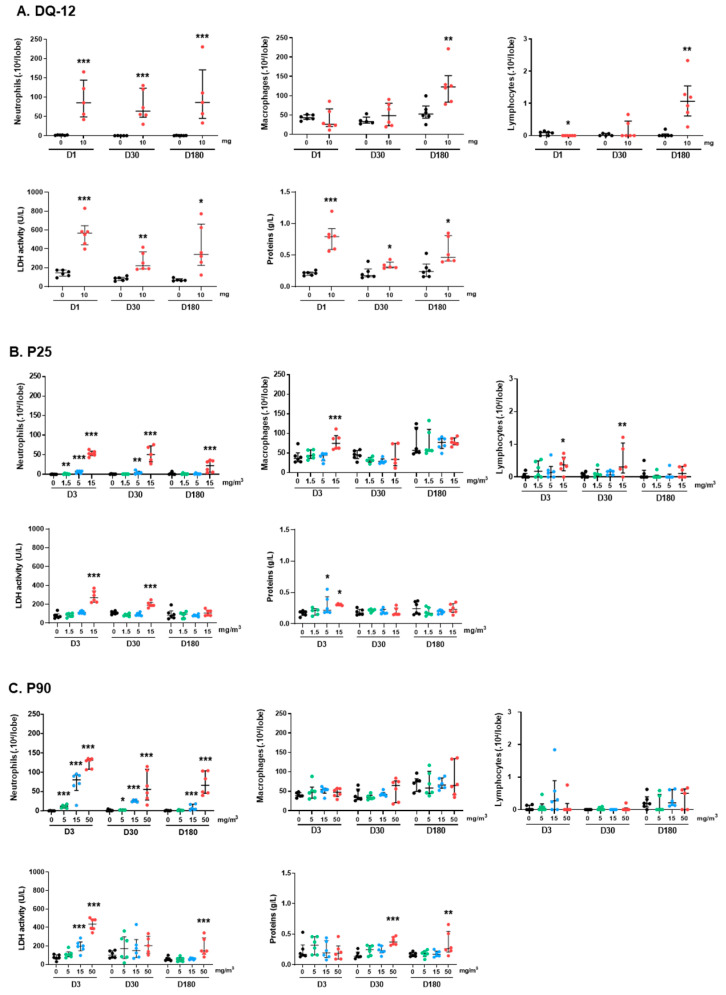

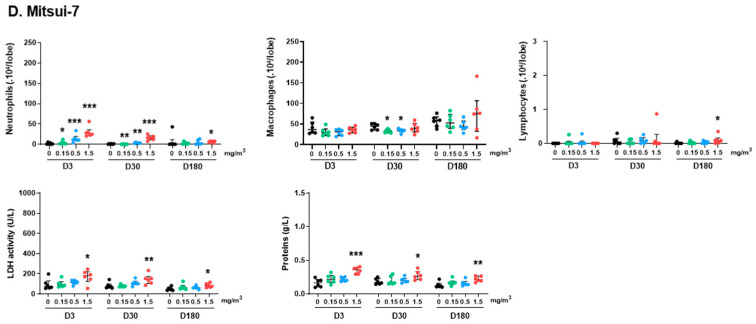
Exposure to DQ-12, P25, P90, and Mitsui-7 induces lung inflammation. Cell content (neutrophils, macrophages, and lymphocytes) and biochemical parameters (LDH activity and protein content) were analyzed in BALF at the times indicated following exposure to (**A**) 10 mg/rat of DQ-12; (**B**) 1.5, 5, or 15 mg/m^3^ of P25; (**C**) 5, 15, or 50 mg/m^3^ of P90; (**D**) 0.15, 0.5, or 1.5 mg/m^3^ of Mitsui-7. Data are expressed as median [Q1; Q3]. * *p* < 0.05, ** *p* < 0.01, *** *p* < 0.001 significantly different from the control (*n* = 6).

The cytology of the bronchoalveolar lavage (BAL) was analyzed following intratracheal instillation of DQ-12. An increase in the neutrophil influx was observed from 1 day after exposure (D1), and remained elevated up to 180 days (Figure 2A). This influx was accompanied by pulmonary cytotoxicity and alveolar permeability, revealed by an increase in lactate dehydrogenase (LDH) activity and protein content at the three post-exposure times. The numbers of macrophages and lymphocytes were comparable to those counted in control animals at D1 and D30; however, at D180, after exposure to DQ-12, their numbers increased. For P25 (Figure 2B), P90 (Figure 2C), and Mitsui-7 (Figure 2D), a dose-dependent increase in neutrophils was observed 3 days after the end of exposure. Although neutrophil numbers dropped over time, they were still significant at D180 after exposure to the highest dose of all three particles. Exposure to P90 or Mitsui-7 slightly modified the number of macrophages and lymphocytes at all doses and post-exposure time points. In contrast, inhalation of 15 mg/m^3^ of P25 only induced an increase in macrophage numbers at D3, and an increase in lymphocyte numbers at D3 and D30. LDH activity was also induced 3 and 30 days after exposure to the high dose of P25, whereas an increase in protein content was measured only at D3 after the end of exposure to 5 and 15 mg/m^3^ of this NP (Figure 2B). Exposure to 15 and 50 mg/m^3^ of P90 also induced LDH activity at D3. At D180, only the high dose was still associated with increased LDH activity. The protein content measured following exposure to the high dose of P90 increased between 30 and 180 days (Figure 2C). Similarly, only the highest dose of Mitsui-7 affected LDH activity and protein content, with increases observed at the three post-exposure times (Figure 2D).

Respiratory parameters were measured before, during, and after inhalation. Overall, no differences were observed between controls and exposed animals. However, two parameters were significantly decreased following P90 exposure: the peak expiratory flow and relaxation time (Supplementary Table S2).

#### 2.1.2. Cytokines and Genotoxicity

**Figure 3:**
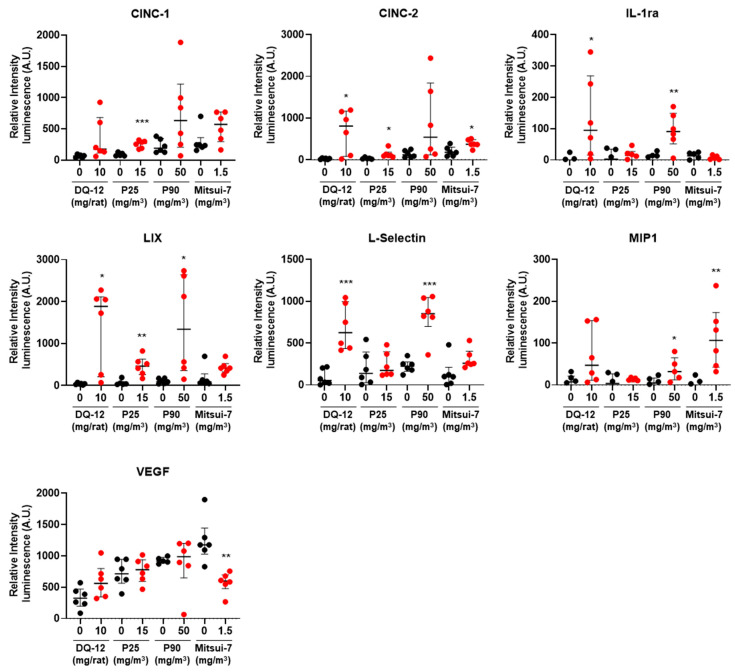
Exposure to DQ-12, P25, P90, and Mitsui-7 increases cytokine protein levels in BALF. Protein expression in BALF was analyzed at D180 after exposure to DQ-12, 15 mg/m^3^ of P25, 50 mg/m^3^ of P90, or 1.5 mg/m^3^ of Mitsui-7. Proteins for which levels were significantly modified are presented as median [Q1; Q3]. * *p* < 0.05, ** *p* < 0.01, *** *p* < 0.001 significantly different from the control (*n* = 6).

We then analyzed a panel of cytokines in BAL fluid (BALF) 180 days post exposure to DQ-12 or the highest doses of P25, P90, and Mitsui-7. Exposure to DQ-12, P25, and P90 increased LIX protein levels, and only DQ-12 and P90 increased IL1-ra and L-selectin levels (Figure 3). Higher protein expression levels were measured following exposure to DQ-12 than to P90. Indeed, we observed a 112-fold increase in IL1-ra following the instillation of DQ-12, whereas P90 increased the expression of this protein 10-fold. DQ-12 was found to have double the impact of P90 when examining induction of LIX (34-fold versus 14-fold) and L-selectin (8-fold versus 4-fold). Interestingly, exposure to DQ-12, Mitsui-7, and P25 increased CINC-2 protein levels (33-fold, 2-fold, and 5-fold increases, respectively), exposure to P90 and Mitsui-7 increased MIP-1α protein levels (100-fold and 6-fold increases, respectively), only P25 exposure increased the CINC-1 protein level (about 3-fold increase), and only Mitsui-7 exposure decreased the VEGF protein level (2-fold decrease).

Whatever the nature of the particle, the dose, and the post-exposure time, no major DNA damage was detected in comet assays performed on lung tissue from exposed animals (Supplementary Figure S1A–D). However, there were some differences in BAL cells: low doses of P25 increased DNA damage at D30, whereas the highest dose decreased the level of DNA damage measured on D3 (Supplementary Figure S1F). We also noticed a decrease in DNA damage following DQ-12 exposure (Supplementary Figure S1E), and with the two highest doses of P90 (Supplementary Figure S1G) and Mitsui-7 (Supplementary Figure S1H). Nonetheless, no consistent dose-dependent DNA damage was observed for any of the particles tested. As a positive control (see Supplementary Data), we exposed animals to methyl methanesulfonate with gavage. This treatment produced a clear increase in DNA damage in BALF (22 ± 8% without Fpg and 87 ± 4% with Fpg) and lung tissue (22 ± 7% without Fpg and 88 ± 3% with Fpg). We therefore hypothesized that the effects observed following exposure to NPs could be due to direct interactions between the particles and DNA, which would interfere with DNA migration during electrophoresis.

### 2.2. Histopathology and Phospholipids

**Figure 4:**
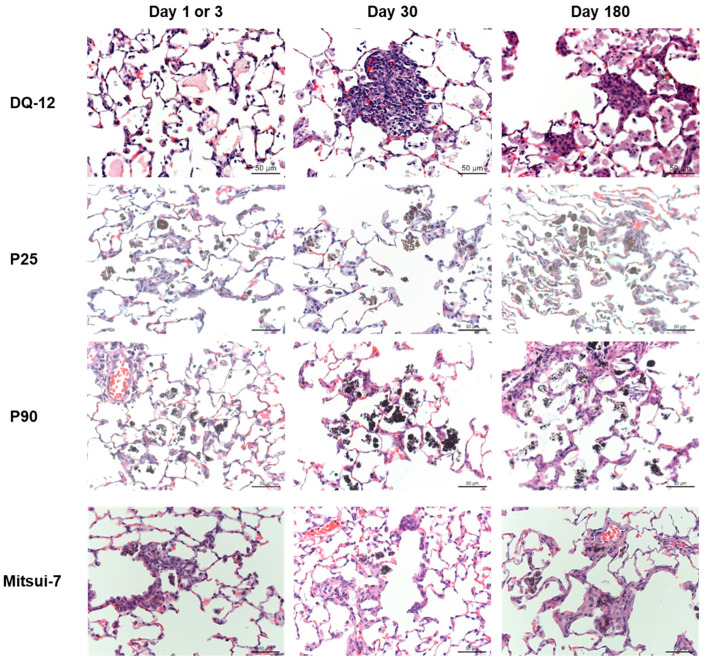
Histopathological analysis of lungs from exposed rats. Histopathological analysis was performed after H&E staining of lung sections from rats exposed to DQ-12, P25, P90, or Mitsui-7. Pictures are representative photomicrographs from the highest dose of particles at each time point (10 mg/rat of DQ-12, 15 mg/m^3^ of P25, 50 mg/m^3^ of P90, and 1.5 mg/m^3^ of Mitsui-7) (*n* = 6).

A histopathological analysis of lungs from animals exposed to DQ-12 showed slight proteinosis at D1, which had increased at D30 and was accompanied by macrophage and lymphocyte aggregates. A specific feature of DQ12 exposure at D30 was the presence of relatively tight aggregates of rounded macrophages; at 180 days, these aggregates also featured collagen deposition. In addition, foci of interstitial thickening with detectable collagen were present at D180 (not affecting the entire lobe). The emergence of these lesions was heterogeneous (Figure 4), but when present, there was a clear progression between D30 and D180.

Exposure to P25 and P90 caused similar lesions. At D3, inhalation of the highest doses of NPs induced interstitial thickening with low levels of collagen deposition. Following P25 and P90 exposure, particle-laden macrophages were observed, which aggregated over time, although granulomas were not present to any great extent (Figure 4). These effects were decreased at low doses (Figure 4). At D180, following exposure to a low dose of P25 or P90, the lungs were almost normal, the medium dose led to minor changes, and with the high dose, the changes were significant and associated with macrophage and lymphocyte aggregates, whereas at D30, the thickening was accompanied by higher collagen levels. At D180, morphological changes were similar to those observed at D30; thus, no progression was observed. Some proteinosis was detected, but not to the extent seen with DQ12.

In Mitsui-7-exposed rat lungs, less material was visible and macrophages did not form aggregates. At D3, in the high-dose group, slight interstitial thickening with morphological changes was observed around the terminal bronchioles, and collagen was detected (Figure 4). At D30, interstitial thickening and collagen were also detected, and histological sections were comparable to those obtained following exposure to P90 and P25. We observed no progression between D30 and D180.

**Figure 5:**
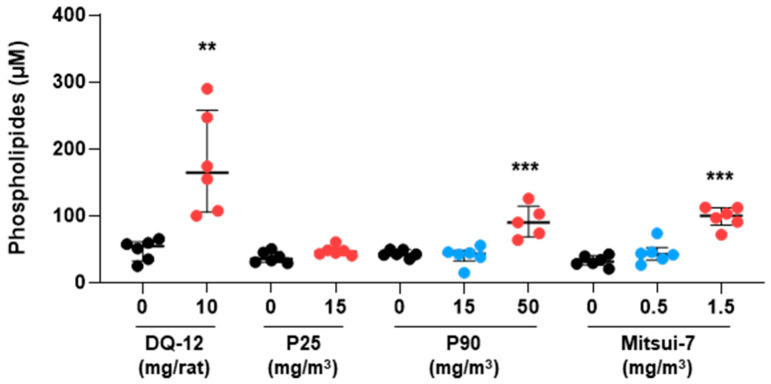
Exposure to DQ-12, P90, and Mitsui-7 induced phospholipid content in BALF. Phospholipid content in BALF was analyzed 180 days after the end of exposure to DQ-12 (10 mg/rat), P25 (15 mg/m^3^), P90 (15 and 50 mg/m^3^), and Mitsui-7 (0.5 and 1.5 mg/m^3^). Data are expressed as median [Q1; Q3]. ** *p* < 0.01, *** *p* < 0.001 significantly different from the control (Printex 90 high dose—*n* = 5, all other conditions *n* = 6).

To complete the assessment of physiopathological changes induced by exposure to the various materials, we measured levels of choline-based phospholipids at D180 following exposure to crystalline silica, the highest dose of P25, and the medium and high doses of P90 and Mitsui-7. We observed a significant increase in phospholipid levels in BALF after exposure to DQ-12 and the high doses of P90 or Mitsui-7, but not following exposure to P25 (Figure 5). Interestingly, the medium dose of P90 and Mitsui-7 did not affect the phospholipid concentration in BALF at D180.

### 2.3. Exposure to Particles Modulated the Gene Expression Profile in Lung Tissue

**Figure 6:**
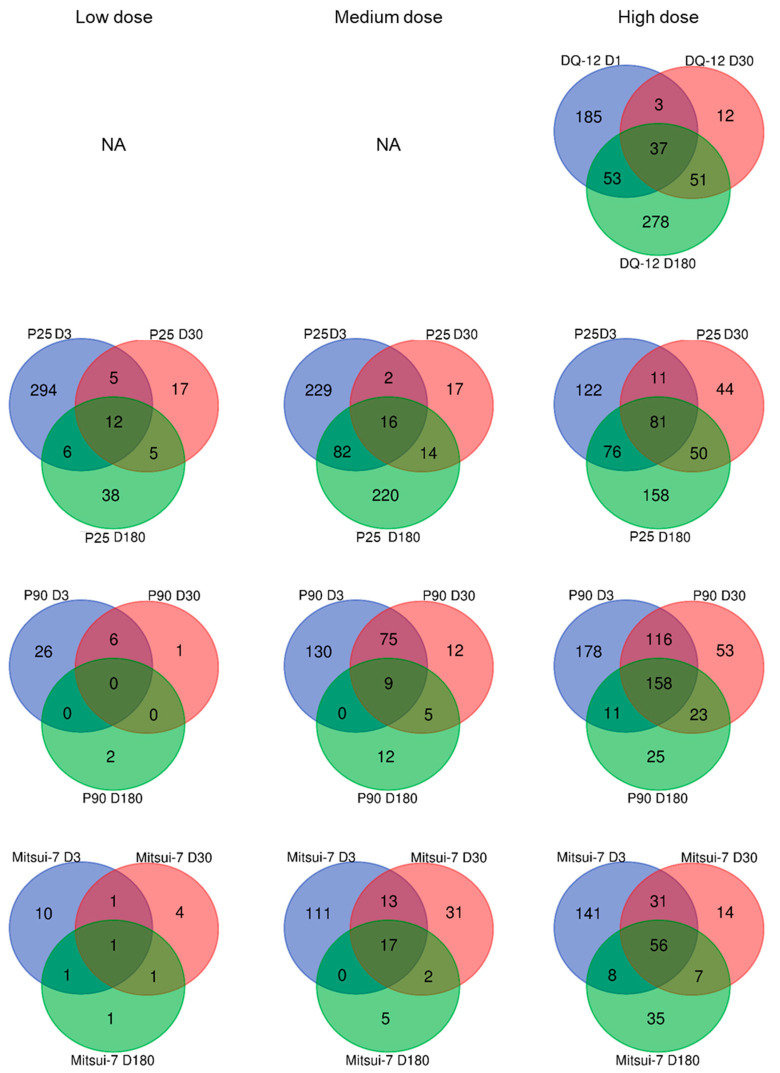
Differentially expressed genes identified in samples from lungs of animals exposed to DQ-12, P25, P90, or Mitsui-7. The number of differentially expressed genes (FC > 2, *p*-value < 0.05) at each time point following exposure to DQ-12, P25, P90, or Mitsui-7 is presented in the Venn diagram. Low dose: 1.5 mg/m^3^ of P25, 5 mg/m^3^ of P90, and 0.15 mg/m^3^ of Mitsui-7; medium dose: 5 mg/m^3^ of P25, 15 mg/m^3^ of P90, and 0.5 mg/m^3^ of Mitsui-7; high dose: 15 mg/m^3^ of P25, 50 mg/m^3^ of P90, and 1.5 mg/m^3^ of Mitsui-7. DQ-12: 10 mg/rat. NA: Not applicable.

Intratracheal instillation of DQ-12 resulted in 278 DEGs at D1, 103 at D30, and 420 at D180 (Table 1). A similar V-shaped profile, with a decrease between 1 and 30 days followed by an increase between 30 and 180 days, was associated with P25 exposure at all doses (Table 1). It is interesting to note that the number of DEGs was similar at D3, regardless of the P25 dose, whereas a dose-dependent increase in DEGs was observed at D30 and D180. A dose-dependent increase in the DEGs commonly modulated at the three times was also observed following inhalation of P25, whereas the number of DEGs specifically affected at D180 was similar for the medium and high doses (Figure 6). Following exposure to P90 and Mitsui-7, a different profile was observed, with the number of DEGs showing a dose-dependent response that decreased over time whatever the exposure concentration (Table 1). Although few genes were differentially affected with the low doses, some DEGs were common between all three time points (Figure 6). Thus, after exposure to 50 mg/m^3^ of P90, a total of 158 genes were affected at all three time points, whereas after exposure to 1.5 mg/m^3^ of Mitsui-7, expression of 56 genes was altered at the three times.

### 2.4. Gene Expression Signature Predictive of Adverse Outcomes

**Figure 7:**
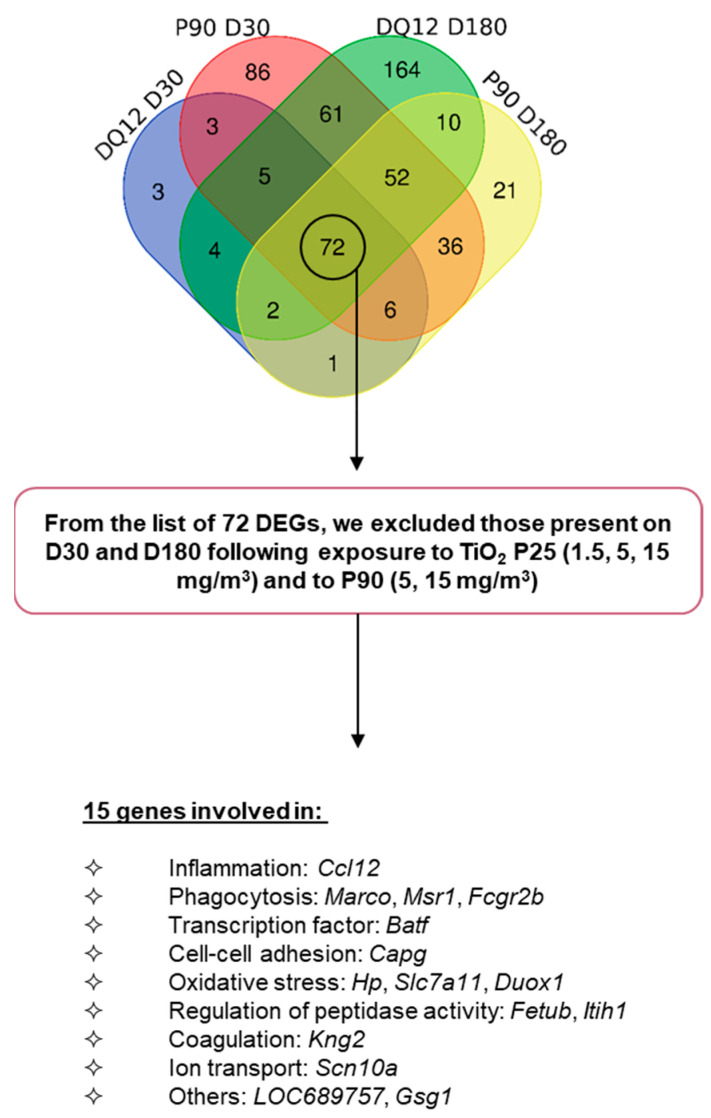
Differentially expressed genes associated with phospholipid accumulation following exposure to spherical particles. The transcriptome was analyzed at the three time points following exposure to DQ-12, P25, and P90. FC > 2, *p*-value < 0.05. From the list of 72 DEGs in common following exposure to DQ-12 and the high dose of P90 at D30 and D180, 15 were not differentially expressed at D30 and D180 following exposure to P25 (1.5, 5, 15 mg/m^3^) or P90 (5, 15 mg/m^3^). DEGs: differentially expressed genes.

As we had recorded phospholipid accumulation in BALF 180 days after exposure to DQ-12, and to a lesser extent following exposure to the highest dose of P90 but not P25, we sought to identify genes that were differentially expressed at a shorter time point, which could serve as predictive markers of AO. Shortly after the end of exposure (D1 and D3), a pleiotropic response to particle exposure was observed in the form of acute inflammation. At D30, either recovery or a pathological process was engaged. Hence, at this post-exposure time, genes that were specifically differentially expressed in conditions leading to long-term adverse effects could represent early markers of pathological effects. To identify these genes, we first compared DEGs common between DQ-12 and the two highest doses of P90, at D30 and D180 (Figure 7). From the 72 genes identified, we excluded those that were deregulated at D30 and D180 following exposure in conditions not associated with lung AO (phospholipid accumulation in BALF)—i.e., low- and medium-doses of P90, and the three doses of P25. Following this exclusion, 15 genes remained that could predict the long-term adverse effect. An analysis of the functions of these genes revealed them to be involved in inflammation (Ccl12), phagocytosis (Marco, Msr1, Fcgr2b), transcription (Baft), cell–cell adhesion (Capg), oxidative stress (Hp, Slc7a11, Duox1), regulation of peptidase activity (Fetub, Itih1), coagulation (Kng2), ion transport (Scn10a), and other pathways (LOC689757, Gsg1) (Figure 7). The fold changes for the DEGs identified 30 and 180 days after exposure to DQ-12 and the high dose of P90 are presented in Table 2.

**Figure 8:**
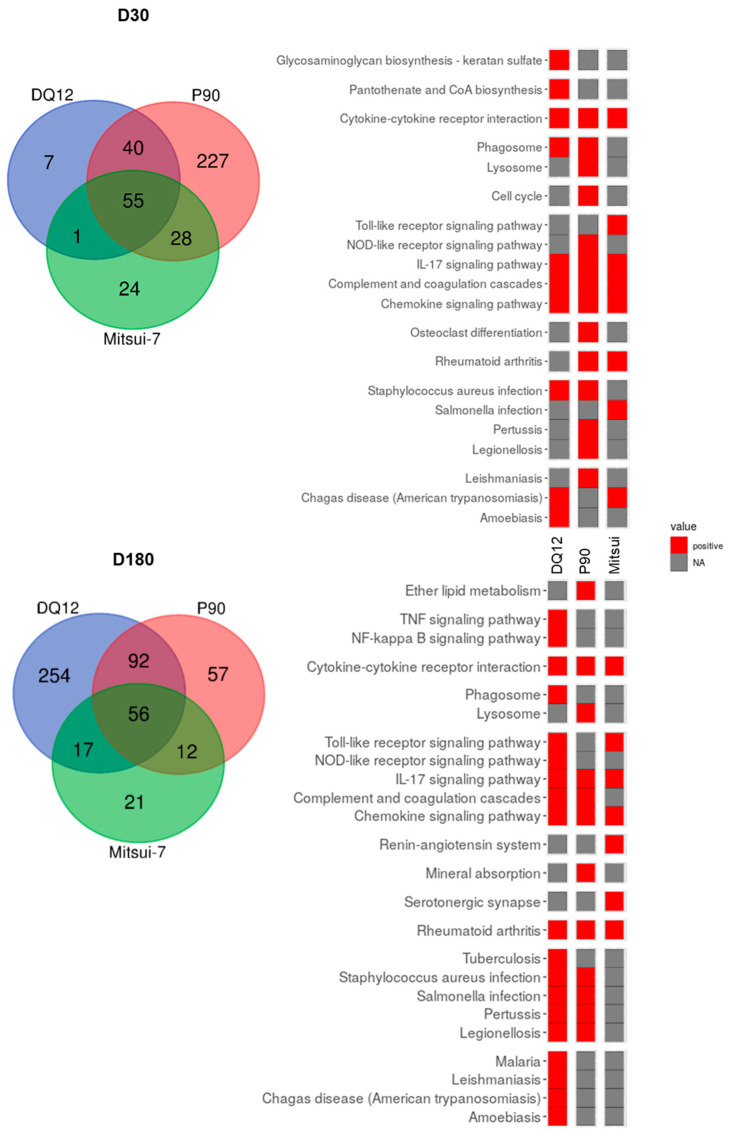
Venn diagram and heat map of signaling pathways deregulated following exposure to NPs. DEGs identified in D30 and D180 lung samples from rats exposed to DQ-12 and the high doses of P90 and Mitsui-7 were compared and presented as a Venn diagram. Heat maps were generated for differently expressed genes. The upper panel shows data from day 30 and the lower panel shows data from day 180. The color of the boxes represents the level of expression of the majority of genes involved in each pathway. Red boxes: overexpression in exposed compared to control animals, gray boxes: no differences between test and control animals.

We compared this list of 15 DEGs to the genes modulated following exposure to Mitsui-7 at the same time points. Only LOC689757 was overexpressed at the two time points following Mitsui-7 exposure. Therefore, we compared the DEGs for rats exposed in conditions leading to increased phospholipid content. The Venn diagrams at D30 and D180 indicate that there were more DEGs in common between DQ-12 and P90 than between DQ-12 and Mitsui-7 (Figure 8). To identify specific pathways triggered by spherical (DQ-12 and P90) or fibrous (Mitsui-7) materials, we compared the deregulated pathways identified at D30 and D180. “Phagosome” and “Complement and coagulation cascades” were specifically induced with exposure to spherical particles at D30 and D180, respectively. In contrast, Mitsui-7 inhalation specifically induced the “Toll-like receptor signaling pathway” at D30 and the “Renin–angiotensin system” at D180.

## 3. Discussion

Exposure to NPs raises concerns about safety in workplaces, where workers can be exposed mainly through the inhalation of particles during handling or manufacturing. Therefore, toxicological analyses of NPs are necessary, but faster and more reliable assays are required to reduce the time it takes to assess hazards and to ensure the safety of products available on the market. High-throughput and high-content methods, such as transcriptomics, are promising approaches when seeking to understand the underlying toxicity mechanisms triggered by NP exposure. These methods can also be useful for the identification of markers, which could serve to predict potential toxic effects of NPs [21,22,23]. The aim of this study was to compare transcriptomic profiles for three classes of NPs—P90, P25, and Mitsui-7. DQ-12 was used as a positive control.

The experiments were performed with female rats. We have previously shown that there was no significant difference in terms of inflammation between males and females following inhalation of nanoparticles [24]. However, in transcriptomic assays, it has been reported that ozone induced more changes in the expression of genes linked to inflammation in female mice than in males [25]. The authors of that study also observed a higher inflammatory response in female rodents. Taken together, these results suggest that the findings presented here, obtained with female rats, could be generalized to males. Although the differences between male and female rats are not particularly striking, females appear to be the more sensitive animal model. From an occupational point of view, the main priority is to protect the most sensitive population, and it is therefore relevant to perform this type of test on female animals.

For our tests, we used a reference material (crystalline silica DQ-12) that is known to induce pulmonary fibrosis and other adverse effects such as lipoproteinosis and phospholipid accumulation in BALF [26], and compared its effects to those of inhaled CB P90 and TiO_2_ P25. Effects were compared based on both conventional and molecular approaches. The molecular analyses identified a set of genes that might be linked to the induction of phospholipid content, with effects detected at earlier time points than with histopathological changes.

Animals were exposed to DQ-12 with intratracheal instillation as a standard positive control. For the inhalation exposure to P25 and P90, we used two distinct aerosolization methods that are well suited to the materials. Thus, we used a SAG410/U aerosol generator for P90 to generate up to 50 mg/m^3^ of the nanostructured aerosol. For P25, we used a RBG1000G generator. In these conditions, it was impossible to exceed 15 mg/m^3^ without affecting the stability of the aerosol and moving away from the OECD guidelines relating to an agglomerate size and distribution [27]. Mitsui-7 was aerosolized with an acoustic generator, as in other studies with carbon nanotubes [28,29]. The maximum airborne concentration achievable providing stable aerosol characteristics over time depends on the dustiness of the material [30]; with Mitsui-7, it was 1.5 mg/m^3^.

Two exposure scenarios were used depending on the product, intratracheal instillation and nose-only inhalation. In a previous study, we demonstrated a similar dose–response relationship for carbon nanotube exposure, in terms of a pulmonary inflammatory response, following sub-acute inhalation and a single intratracheal instillation [29]. Based on these results, we concluded that the inflammatory response triggered by the two exposure scenarios was broadly equivalent, but that the surface area of the nanotube deposited in the lung had to be taken into account. Here, the effects of DQ-12, administered with intratracheal instillation, are necessary to be considered in the context of the exposure technique. However, as DQ-12 was only used as a positive control for pulmonary fibrosis at a single dose, this remark does not alter the interpretation of our results.

### 3.1. Conventional Approach

Exposure to the crystalline silica DQ-12 induced inflammation, alveolar permeabilization, and increased lung weight in the short and long term, to a similar extent, as shown here and elsewhere [15,16,17,18]. The neutrophil influx persisting at D180 is in accordance with the elevated levels of certain cytokines, detected in protein assays (IL1-ra, LIX, L-selectin, CINC-2). In addition, exposure to DQ-12 caused pulmonary alveolar lipoproteinosis, in line with the increase in BALF phospholipid content [16].

We showed that nose-only exposure to TiO_2_ P25—a low-toxicity, low-solubility material—induced lung inflammation with alveolar permeabilization and an increase in lung weight. This lung damage was reversible, and the neutrophil influx reduced 180 days after exposure to P25.

No significant pulmonary alveolar phospholipid accumulation was observed following exposure to P25. Due to the lower aerosol concentration, the less pronounced effect of this NP could be due to a lower lung-deposited surface area.

Nose-only exposure to a high dose (50 mg/m^3^) of CB P90 induced lung inflammation, alveolar permeabilization, and an increase in lung weight. This lung damage was still visible 180 days after exposure to the highest dose of P90, and was accompanied by an increase in protein levels of the cytokines IL1-ra, LIX, L-selectin, and MIP-1α. Similar effects on cytokine expression have been reported after instillation exposure by Ernst and collaborators [16]. Following exposure to P90, we did not notice a significant increase in protein content in BALF at D180; however, the phospholipid content was increased, without significant lipoproteinosis. These different types of lung damage could all contribute to CB-induced fibrosis, as shown recently [20,31]. Due to the low toxicity of P90, the adverse effects observed could be associated with the very high level of deposition of an NP in the lungs and the development of a lung overload mechanism [32].

The high dose of Mitsui-7 (1.5 mg/m^3^) induced pulmonary changes—inflammation, cytotoxicity, and interstitial thickening—associated with collagen deposition and increased BALF phospholipid content. Quantitatively, these perturbations were comparable to those induced by spherical particles, but the qualitative histological analysis revealed differences in line with observations reported by Knudsen et al. [33] following exposure to long and thick MWCNTs.

### 3.2. Molecular Approach

In order to identify genes that could be used as earlier markers of lung AO, we compared DEGs from the transcriptomic analysis following exposure to spherical particles (DQ-12—10 mg/rat; P25—1.5, 5, and 15 mg/m^3^; P90—5, 15, and 50 mg/m^3^). First, we identified materials and their concentrations inducing phospholipid accumulation at 180 days after exposure. This marker could be considered a good marker of long-term AO such as fibrosis, although no such pathological change was detected during the histopathological examination of lungs from P90-exposed animals [19,20]. We then identified DEGs at 30 and 180 days after exposure, which were specific to phospholipid accumulation conditions, and finally we determined and proposed a list of DEGs detectable at 30 days after exposure.

Shortly after exposure (1 or 3 days, depending on the condition), lung inflammation was the most striking effect since it is the first step in the organism’s defense. In line with this, a considerable number of inflammation-related genes were found to be differentially expressed. Based on this result, it can be considered that the DEGs found at this early time were more a consequence of the acute effects of exposure to a material and the inflammatory response triggered by the foreign bodies (i.e., (nano)particles). Thirty days after the end of exposure, we considered that, depending on the toxicity of the material, either the pulmonary tissue would have recovered from injury, or the damage to the lungs would be too severe and the deleterious effects would be evolving toward the development of AO. Based on this reasoning, we considered that certain DEGs at D30 might be relevant markers for long-term effects. To identify these candidate markers, we searched for DEGs detected at D30 and D180 in animals exposed in conditions that induced lung AO (i.e., DQ-12—10 mg/rat and P90—50 mg/m^3^); 72 DEGs were identified. From this list, we eliminated the DEGs detected in samples exposed to conditions that did not induce lung AO (i.e., P90—5 and 15 mg/m^3^; P25—1.5, 5, and 15 mg/m^3^) at the same post-exposure times. We were left with 15 genes: *Ccl12*, *Marco*, *Msr1*, *Fcgr2b*, *Batf*, *Capg*, *Hp*, *Slc7a11*, *Duox1*, *Fetub*, *Itih1*, *Kng2*, *Scn10a*, *LOC689756*, and *Gsg1*.

In order to verify that our DEG list can be generalized to other studies, we compared our data with other publications on silica or lunar dust-induced lung fibrosis [34,35,36,37,38] in rats following inhalation or intratracheal instillation (Table 3). One gene, *Fetub*, was differentially expressed in all papers and three others (*Fcgr2b*, *Hp*, and *Capg*) were differentially expressed in five out of six transcriptomic analyses. Then, these genes could be considered as good candidates in the identification of early predictors of particle-induced lung fibrosis in rat experimental models in order to reduce the time and the number of animals used in such experiments. However, one might argue that such candidate genes are only representative of an in vivo study and might not relate to human lung fibrosis. Then, in order to strengthen the predictivity of the modified expression of these genes, we compared this list to that of deregulated genes in patients suffering from silicosis. Pang et al. used transcriptomics to compare the global gene expression profile of 7 healthy and 10 silicosis patients [39]. They identified 1169 up-regulated genes and 1436 down-regulated genes in silicosis patients. Among those, only two were common with our list: *Fcgr2b* and *Capg*. In addition, these deregulated genes were not found in idiopathic pulmonary fibrosis nor chronic obstructive pulmonary disease patients. According to the Human Protein Atlas (https://www.proteinatlas.org/, accessed on 10 March 2022), *Fcgr2b* encodes for “the Receptor for the Fc region of complexed or aggregated immunoglobulins gamma. Low affinity receptor. Involved in a variety of effector and regulatory functions such as phagocytosis of immune complexes and modulation of antibody production by B-cells”, and *Capg* for the “Calcium-sensitive protein which reversibly blocks the barbed ends of actin filaments but does not sever preformed actin filaments. It may play an important role in macrophage function”. However, no clear link between the expression of these genes and fibrosis has been established.

Unfortunately, none of the 15 genes we identified with spherical (nano)particles were deregulated after Mitsui-7 exposure in our study. However, Mitsui-7 did modify the expression of genes involved in the inflammatory response, with effects persisting even after 30 and 180 days. These effects are related to the influx of neutrophils into the lungs. Persistent inflammation could be responsible for the development of lung pathologies. Even if we did not observe pulmonary fibrosis after Mitsui-7 inhalation, histopathological modifications, such as interstitial thickening, associated with phospholipid accumulation could lead to long-term AO. In addition, it is now well established that pulmonary exposure to Mitsui-7 induces lung diseases such as cancer [40] and fibrosis [41]. Snyder-Talkington et al. [41] identified a “fibrosis list” of specific DEGs in mice after exposure to Mitsui-7 (carbon nanotubes). None of the DEGs identified on our list following exposure to spherical particles were present on this “fibrosis list”. However, interestingly, five of the genes deregulated by Mitsui-7 in our conditions were part of this list (*Il1rn*, *Ccl17*, *Ccl2*, *Cxcl10*, *Mmp12*). This corroboration is interesting as our experimental protocol used a different species and a distinct exposure strategy, and the physiological changes observed did not reach the fibrosis stage.

The DEGs that we identified should be further investigated as predictive genes for long-term fibrosis induced by spherical particles. Tubes/fibers and spherical particles may induce lung AO with a variety of molecular mechanisms. These results are in good agreement with our previous study, where we observed that inhalation of short and thin multi-walled carbon nanotubes (NM-403) forming spherical agglomerates triggered different pulmonary signaling pathways compared to inhalation of long and thick multi-walled carbon nanotubes (NM-401) forming fiber-like agglomerates [42]. Thus, the cellular and biomolecular interactions of spherical particles may differ from those of tubular/fibrous materials, leading to the activation of distinct cellular and molecular responses.

In addition, even though this was not the purpose of our work since it was not designed to identify clinical markers of lung cancer, we compared our gene list with that of patients with lung adenocarcinoma or Non-Small-Cell Lung Cancer. No common differently expressed gene was noticed between the different studies [43].

As the conclusion to this study, we present a list of differentially expressed genes representing potential candidates that could be associated with AO following exposure to spherical nanoparticles. According to the literature, two of them, *Fcgr2b* and *Capg*, might be more promising than the others since they have been identified in other studies on lung fibrosis with rat models or human patients. In addition, our data indicate that carbon nanotubes trigger distinct response pathways. However, the number of types of nanoparticles and carbon nanotubes used remains small. The results presented, although promising, will need to be confirmed by studies involving a larger number of nanoparticles and nanotubes.

## 4. Materials and Methods

### 4.1. Particles

Titanium dioxide (Aeroxide^®^ P25) was purchased from Evonik (Essen, Germany). MWCNT Mitsui-7 (from Mitsui Company (Tokyo, Japan)) and furnace carbon black Printex^®^ 90 (from Evonik^®^ P90) were kindly donated by the National Research Centre for the Working Environment (Copenhagen, Denmark). Crystalline silica DQ-12 was obtained from DMT GmbH & Company KG (Essen, Germany). Because of long-term storage, its surface was reactivated after grinding (10 g) for 15 min at 1400 rpm with a vibratory disc mill RS 200 (Retsch (Haan, Germany)) and tungsten carbide grinding tools. A scanning electron microscopy image of ground DQ-12 particles is presented in Supplementary Figure S2. It should be noted that due to grinding, the particle size and crystallinity of DQ-12 were reduced, and some contamination with tungsten (W) and cobalt (Co) from the grinding tools (<0.6% *w*/*w*) was measured. The main physical and chemical characteristics of the four particles are presented in Table 4.

### 4.2. Experimental Design

Animal experiments were performed in accordance with EC Directive 2010/63/EU. The protocol was approved by the local Ethical Committee and the French Ministry for Research and Higher Education (APAFIS#10052). The animal facility is accredited by the French Ministry of Agriculture (Agreement Number: #D54-547-10). Thirteen-week-old female Sprague Dawley rats (Janvier Labs, Le Genest Saint Isle, France) were randomly distributed between control and exposed groups. Animals were housed by two or three in individually ventilated cages (Tecniplast) maintained in 12 h/12 h light/dark cycles, and they had ad libitum access to food and water.

Animals were exposed to crystalline silica with intratracheal instillation (*n* = 6/group). After intraperitoneal injection of Domitor (0.25 mg/kg) and Ketamine (40 mg/kg), anesthetized animals were instilled with 10 mg/rat of a suspension of DQ-12 in sterile NaCl 0.9% and sonicated for 30 min (Bransonic 250), or with the vehicle alone. A subcutaneous injection of Antisedan (1.25 mg/kg) was used to wake the animals. Animals were euthanized 1, 30, or 180 days after exposure. The experimental procedure for nose-only inhalation exposure was described previously [29]. Briefly, after acclimatization to the restraining tubes, the animals were exposed to filtered air, P25, P90, or Mitsui-7 5 days/week for 4 weeks (*n* = 6/group). Target concentrations of 15 mg/m^3^ of P25, 50 mg/m^3^ of P90, and 1.5 mg/m^3^ of Mitsui-7 6 h/day were applied for the high-dose groups. Based on the C × t protocol (concentration × time) [45], the medium-dose groups were exposed to the same concentrations for 2 h/day; this corresponds to a 6 h-equivalent concentration of 5 mg/m^3^ of P25, 15 mg/m^3^ of P90, and 0.5 mg/m^3^ of Mitsui-7. Similarly, the low-dose groups were exposed for 36 min/day, corresponding to a 6 h-equivalent concentration of 1.5 mg/m^3^ of P25, 5 mg/m^3^ of P90, and 0.15 mg/m^3^ of Mitsui-7. Regardless of the duration of exposure to nanoaerosols, all animals remained in the restraining tubes for 6 h/day and were exposed to filtered air when not exposed to particles. Animals were euthanized 3, 30, or 180 days after exposure. The P25 and P90 aerosols were generated with a rotating brush RBG1000 (PALAS, Karlsruhe, Germany) and SAG410/U (TOPAS, Dresden, Germany), respectively. The Mitsui-7 aerosol was produced by an acoustic generator (IEStechno, Morgantown, WV, USA). The target and actual mass concentrations are presented in Table 5 alongside the main characteristics of the aerosols generated: number concentration, mass median aerodynamic diameter (MMAD), count median aerodynamic diameter (CMAD), associated geometric standard deviation (GSD), and aerosol effective density [24]. The number and mass size distribution of the aerosols are shown in Supplementary Figure S3. Table 6 summarizes the mode of exposure, dose or concentration of the materials used, and post-exposure time points.

### 4.3. Necropsy and Tissue Sampling

Tissues were collected following deep anesthesia with intraperitoneal injection of Xylasine (10 mg/kg)–Ketamine (75 mg/kg), followed by exsanguination through the abdominal aorta. Bronchoalveolar lavage (BAL) was performed on the left lung as previously described [29]. BALF cytology was analyzed on cytospin preparations after May–Grünwald–Giemsa staining; 500 cells/animal were analyzed. Caudal lung lobes were embedded in paraffin for the histological analysis, and accessory lobes were collected in a RNAlater solution to perform transcriptomic analyses.

### 4.4. Cytokine/Chemokine Detection

The relative levels of 29 cytokines and chemokines in BALF (*n* = 6) were assessed using the Proteome ProfilerTM Rat Cytokine Array Kit, panel A (ARY008—R&D Systems), according to the manufacturer’s recommendations. The rat cytokine array membrane was blocked with Array Buffer 6 for 1 h at room temperature. In the meantime, 0.5 mL of BALF samples was mixed with Array Buffer 4, and the final volume was adjusted by adding Array Buffer 6 (up to 1.5 mL). Samples were then incubated for 1 h at room temperature with a Detection Antibody Cocktail followed by incubation with a Rat Cytokine Array membrane overnight at 4 °C. Membranes were washed and incubated with a Streptavidin-HRP solution for 30 min at room temperature. The cytokine/chemokine spots were detected by applying a chemiluminescent detection reagent, and analyzed with a Bio-Rad ChemiDoc™ MP imaging system (Image Lab Software v.6.0). The mean chemiluminescence was normalized relative to reference spots on the same membrane after background correction.

### 4.5. Histological Analysis

Right caudal lung lobes were slowly filled and fixed with 4% neutral buffered formaldehyde (Sigma Aldrich, St. Louis, MI, USA). Following dehydration, they were embedded in paraffin. Then, tissue blocks were sectioned and slides were stained using hematoxylin and eosin or Sirius red prior to analysis under bright-field microscopy.

### 4.6. Phospholipid Content Analysis

The level of phospholipids in BALF collected at D180 from animals exposed to DQ-12, a high concentration of P25, or the medium or high concentration of P90 and Mitsui-7 was determined by measuring choline-based phospholipids using a phospholipid assay kit according to the manufacturer’s recommendations (MET-5085, Cell Biolabs, Inc., San Diego, CA, USA). Standard (50 µL) and diluted samples were incubated with 50 µL of a detection reagent (hydrolyzing enzyme, oxidoreductase, HRP, and florescent probe) for 30 min at 37 °C, protected from light. Each sample was incubated with and without an hydrolyzing enzyme, to allow subtraction of the background due to the basal choline level. Fluorescence was measured using an excitation wavelength of 560 nm and an emission wavelength of 590 nm. To determine the level of phospholipids within samples, the mean fluorescence of each sample was compared to the fluorescence obtained for a phospholipid standard curve. The blank and basal levels of choline were subtracted from all the values recorded for the samples.

### 4.7. Transcriptomic Analysis of Lung Tissue

Accessory lung lobes from six animals per group collected in the RNAlater solution (Sigma Aldrich) were disrupted using a gentleMACS™ Dissociator (Miltenyi Biotech, Bergisch Gladbach, Germany). Total RNA was extracted and purified with the Nucleospin RNA Midi Kit^®^ (Macherey-Nagel, Hoerdt, France). RNA purity, determined with a NanoPhotometer^®^ spectrophotometer (IMPLEN), was assessed. A260/A280 ratios of around 2 were obtained for all samples. RNA integrity values between 6.6 and 9.3 were obtained by an analysis with an RNA 6000 Nano Assay kit on a 2100 Bioanalyzer system (Agilent Technologies, Santa Clara, CA, USA). Total RNA was stored at −80 °C until further use. RNA (100 ng) was reverse transcribed into cDNA. cDNA labeled with Cyanine 3-CTP (Low Input Quick Amp Labeling kits—Agilent Technologies) was then purified using an RNeasy^®^ Plus Mini kit (Qiagen, Germantown, MD, USA). Labeled cDNAs were hybridized for 17 h at 65 °C on Agilent G4858A SurePrint G3 Unrestricted GE 8*60K microarrays v2 (Agilent Technologies). Slides were washed before scanning on an Agilent G2505C microarray scanner at a 3 µm resolution. Data were extracted using Agilent Feature Extraction software version 12.0.1.1. Microarray data were uploaded to the NCBI Gene Expression Omnibus (GEO) database under the accession numbers GSE158903 for DQ-12, GSE160117 for P25, GSE160155 for P90, and GSE160175 for Mitsui-7 (http://www.ncbi.nlm.nih.gov/geo).

### 4.8. Differential Gene Expression Analysis

To differentially analyze results for control and exposed rats, we used the eUTOPIA tool (solUTion for Omics data Preprocessing and Analysis, https://github.com/Greco-Lab/eUTOPIA, accessed on 21 September 2022) [46], using a protocol described previously [47]. Briefly, after performing quality controls and probe filtering based on quantiles, all data were normalized by applying a quantile method. Batch correction was also performed. Finally, a differential analysis was carried out based on the Limma model and *p*-value adjustment with the Benjamini–Hochberg method. Genes with a Fold-Change (FC) of at least 2 (or log FC = ǀ1ǀ) and an adjusted *p*-value < 0.05 when comparing data for exposed versus control rats were considered significantly differentially expressed. The filtration strategy of differentially expressed genes is presented in a remark diagram in Supplementary Figure S4. Heatmaps were generated using the FunMappOne tool [48] (https://github.com/Greco-Lab/FunMappOne, accessed on 22 September 2022).

### 4.9. Statistical Analysis

Data are expressed as median [Q1; Q3] with individual values, with the first (Q1) and third (Q3) quartile corresponding to 25 and 75% of the scores, respectively. For the physiological data, except the transcriptome analysis, a linear model was used and adjusted for the time and treatment and associated with a Fisher test (nlme package, R, Pinheiro, Bates, DebRoy, Sarkar and the R Development Core Team 2013. nlme. R package version. 3.1-137; www.r-project.org, accessed on 1 February 2022).

### 4.10. Supplementary Methods

Respiratory parameters and comet assay methods are detailed in Supplementary data (under Table S2 and Figure S1, respectively).

## 5. Conclusions

Lung inflammation is the first line of defense of the organism following pulmonary exposure. However, persistent inflammation can be the starting point for the development of an adverse outcome. In this work, we identified 15 genes that were differentially expressed at an early stage in the development of pathological changes following exposure to spherical particles. These pathological changes might potentially develop into lung fibrosis. This group of 15 genes could be used as predictive markers to reduce the time required to assess the toxicological properties of an NP. Our results suggest that histopathological changes in the lungs induced by tube/fiber and spherical particles may be caused by distinct molecular mechanisms.

## Figures and Tables

**Table 1:** Number of differentially expressed genes following exposure to DQ-12, P25, P90, or Mitsui-7.

		Day 1 or 3	Day 30	Day 180
DQ-12	10 mg/rat	278	103	419
P25	1.5 mg/m^3^	317	39	61
	5 mg/m^3^	329	49	332
	15 mg/m^3^	290	186	365
P90	5 mg/m^3^	32	7	2
	15 mg/m^3^	214	101	26
	50 mg/m^3^	465	350	217
Mitsui-7	0.15 mg/m^3^	13	7	4
	0.5 mg/m^3^	141	63	24
	1.5 mg/m^3^	236	108	106

Genes were defined as differentially expressed when FC > 2 and adjusted *p*-value < 0.05.

**Table 2:** Fold changes for the 15 DEGs identified following exposure to DQ-12 and the high dose of P90 (30 and 180 days post exposure).

	DQ-12		P90	
	D30	D180	D30	D180
*Ccl12*	3.3	6.5	3.5	2.1
*Marco*	2.1	2.4	2.3	2.4
*Msr1*	2.2	2.5	3.4	2.2
*Fcgr2b*	2.3	4.1	4.3	2.7
*Batf*	2.0	2.0	3.0	2.4
*Capg*	2.1	3.0	2.9	2.3
*Hp*	2.0	2.7	2.4	2.1
*Slc7a11*	2.5	4.6	2.6	3.0
*Duox1*	2.1	2.4	2.1	2.3
*Fetub*	2.1	2.3	2.8	2.3
*Itih1*	2.9	3.2	4.1	2.6
*Kng2*	2.4	3.1	2.5	2.4
*Scn10a*	2.8	3.1	3.5	2.9
*LOC689757*	2.0	7.0	4.1	2.9
*Gsg1*	2.9	7.5	3.4	2.4

**Table 3:** Comparison of DEG list from the literature.

	DEGs from the Literature				
Gene List	Sellamuthu et al., 2013 [36]	Umbright et al., 2017 [37]	Cai et al., 2021 [35]	Bo et al., 2022 [34]	Zhang et al., 2023
*Ccl12*			x	x	
*Marco*			x		
*Msr1*			x	x	
*Fcgr2b*	x		x	x	x
*Baft*	x				
*Capg*	x		x	x	x
*Hp*	x	x	x		x
*Slc7a11*			x	x	
*Duox1*	x			x	
*Fetub*	x	x	x	x	x
*Itih1*	x		x		
*Kng2*				x	x
*Scn10a*			x		
*LOC689757*				x	
*Gsg1*					
**Total**	**7/15**	**2/15**	**10/15**	**9/15**	**5/15**

X: differentially expressed in the fibrotic lung group as compared to the control one.

**Table 4:** Characteristics of DQ-12, Mitsui-7, P25, and P90.

		Diameter (nm)	SBET ^d^ (m^2^/g)	Purity (%)	Crystallinity Rate (%)
DQ-12 ^a^ After grinding	Crystalline silica	100–1000 ^b^ 220 ^c^	10.3 ± 2.6 ^e^	Si: 99.4% Co: 808 ± 48 µg/g W: 4829 ± 87 µg/g ^e^	66 ^e^
P25	Titanium dioxide	Anatase: 21 ± 1.5 Rutile: 40 ± 1.5	55	99.9	87% anatase and 13% rutile crystallites
P90	Carbon black	14	316	~99	
Mitsui-7	Carbon nanotube	Diameter: 88 ± 5 nm Length: 5.0 ± 4.5 µm	15	99	

^a^ Obtained from DQ-12 raw material (diameter = 1–3 µm; SBET = 3.5 ± 0.9 m^2^/g; crystallinity rate = 90%; purity = 99.9%). ^b^ MEB observation. ^c^ SBET equivalent diameter, assuming spherical particles and silica density = 2.65 g/cm^3^ [44]. ^d^ SBET: Specific surface area determined with the Brunauer, Emmett, and Teller method. ^e^ In-house measurements; crystallinity determined with DRX.

**Table 5:** Characteristics of Mitsui-7, P25, and P90 aerosols.

	Target Concentration (mg/m^3^)	Actual Mass Concentration (mg/m^3^) ^a^	Number Concentration (Particles/cm^3^) ^b^	MMAD (µm) ^c^	CMAD (µm) ^d^	GSDd	Aerosol Effective Density (g/cm^3^) ^e^
P25	15	15.3 ± 4.7	5.1 × 10^4^ ± 1.7 × 10^4^	1.56	0.31	1.72	1.70
	5	5.02 ± 0.39					
	1.5	1.59 ± 0.45					
P90	50	50.1 ± 3.9	3.5 × 10^5^ ± 1.4 × 10^5^	0.94	0.03 and 0.20 ^f^	1.97 and 2.11 ^f^	0.35
	15	15.0 ± 1.24					
	5	4.89 ± 0.39					
Mitsui-7	1.5	1.69 ± 0.49	1.4 × 10^3^ ± 0.5 × 10^3^	1.78	0.40	1.69	0.45
	0.5	0.47 ± 0.15					
	0.15	0.13 ± 0.02					

^a^ The actual mass concentration was derived from four daily samplings and is expressed as the 6 h-equivalent concentration. ^b^ Measured with condensation particle counter (CPC); particle diameter < 3 µm. ^c^ MMAD: mass median aerodynamic diameter. Determined using a DLPI+ cascade impactor. ^d^ CMAD: count median aerodynamic diameter, GSD: geometric standard deviation. Determined from a log-normal fitting of the number size distribution based on either scanning mobility particle sizer (SMPS) or aerodynamic particle sizer (APS) measurements. ^e^ Aerosol effective densities were estimated by merging SMPS and APS number size distributions and assuming spherical particles. ^f^ Bimodal distribution, in number.

**Table 6:** Summary of rat exposures to DQ-12, Mitsui-7, P25, and P90.

Materials	Mode of Administration	Dose/Concentration	Post-Exposure Time Considered	Number of Animals
DQ-12	Instillation	Control, 10 mg/rat	1, 30, and 180 days after exposure	6 per exposure group and post-exposure time
P25	Inhalation (6 h/day, 5 days/week for 4 weeks)	Control, 1.5 mg/m^3 a^, 5 mg/m^3 a^, 15 mg/m^3^	3, 30, and 180 days after exposure	6 per exposure group and post-exposure time
P90	Inhalation (6 h/day, 5 days/week for 4 weeks)	Control, 5 mg/m^3 a^, 15 mg/m^3 a^, 50 mg/m^3^	3, 30, and 180 days after exposure	6 per exposure group and post-exposure time
Mitsui-7	Inhalation (6 h/day, 5 days/week for 4 weeks)	Control, 0.15 mg/m^3 a^, 0.5 mg/m^3 a^, 1.5 mg/m^3^	1, 30, and 180 days after exposure	6 per exposure group and post-exposure time

^a^ 6 h-equivalent concentration created by modulating the time for which animals were exposed to the aerosols produced from a dry powder generator [45].

## Data Availability

The transcriptomic datasets are available in the GEO repository under the numbers GSE158903 for DQ-12, GSE160117 for P25, GSE160155 for P90, and GSE160175 for Mitsui-7 (http://www.ncbi.nlm.nih.gov/geo/).

## Supplementary Materials

The supporting information can be downloaded at: https://www.mdpi.com/article/10.3390/ijms241310890/s1. Reference [49] is cited in the Supplementary Materials.
