# Supplementary material for: Identification of a Gene Signature Predicting (Nano)Particle-Induced Adverse Lung Outcome in Rats
Source: Int J Mol Sci. 2023 Jun 29;24(13):10890. doi: 10.3390/ijms241310890 (PMC10341909; doi:10.3390/ijms241310890)
Supplement: Supplementary file 1 [file ijms-24-10890-s001.zip › ijms-2441821-supplementary.pdf]

Identification of a Gene Signature Predicting (Nano)Particle-Induced Adverse Lung Outcome in Rats

Sarah Amandine Valentino, Carole Seidel, Mylène Lorcin, Sylvie Sébillaud, Henrik Wolff, Stéphane Grossmann, Stéphane Viton, Hervé Nunge, Laura Aliisa Saarimäki, Dario Greco, Frédéric Cosnier, Laurent Gaté

Table S1: Absolute body and lung weights.

Data are expressed as mean ± standard deviation. * $p<0.05$, ** $p<0.01$, *** $p<0.001$ significantly different from controls (n = 6).

			Body weight (g)	Lung weight (g)
DQ-12	D1	0 mg/rat	277.7 ± 12.6	1.27 ± 0.11
		10 mg/rat	262.1 ± 12.8	1.40 ± 0.10
	D30	0 mg/rat	327.7 ± 14.4	1.42 ± 0.11
		10 mg/rat	326.7 ± 16.5	1.67 ± 0.09**
	D180	0 mg/rat	383.6 ± 39.8	1.52 ± 0.08
		10 mg/rat	416.9 ± 49.8	2.03 ± 0.17***
P25	D3	0 mg/m^3	291.5 ± 22.7	1.33 ± 0.08
		1.5 mg/m^3	292.5 ± 13.9	1.38 ± 0.10
		5 mg/m^3	291.7 ± 14.7	1.44 ± 0.07
		15 mg/m^3	291.3 ± 14.8	1.59 ± 0.11***
	D30	0 mg/m^3	338.5 ± 17.7	1.36 ± 0.07
		1.5 mg/m^3	332.5 ± 31.3	1.44 ± 0.14
		5 mg/m^3	329.9 ± 19.0	1.39 ± 0.10
		15 mg/m^3	325.9 ± 20.7	1.56 ± 0.16*
	D180	0 mg/m^3	385.9 ± 37.8	1.48 ± 0.13
		1.5 mg/m^3	401.3 ± 22.0	1.55 ± 0.14
		5 mg/m^3	376.7 ± 39.7	1.48 ± 0.10
		15 mg/m^3	386.5 ± 28.3	1.62 ± 0.10
P90	D3	0 mg/m^3	290.6 ± 8.8	1.29 ± 0.10
		5 mg/m^3	305.4 ± 20.9	1.37 ± 0.06
		15 mg/m^3	295.1 ± 13.7	1.61 ± 0.19***
		50 mg/m^3	298.0 ± 12.1	1.91 ± 0.07***
	D30	0 mg/m^3	322.6 ± 20.9	1.38 ± 0.13
		5 mg/m^3	325.4 ± 11.6	1.50 ± 0.05
		15 mg/m^3	338.4 ± 11.8	1.54 ± 0.08*
		50 mg/m^3	331.2 ± 22.9	1.77 ± 0.17***
	D180	0 mg/m^3	366.0 ± 34.2	1.47 ± 0.06
		5 mg/m^3	372.6 ± 22.7	1.56 ± 0.13
		15 mg/m^3	359.2 ± 23.7	1.51 ± 0.10
		50 mg/m^3	378.4 ± 28.9	1.92 ± 0.21***
Mitsui-7	D3	0 mg/m^3	270.8 ± 12.7	1.30 ± 0.05
		0.15 mg/m^3	296.3 ± 22.7**	1.42 ± 0.10
		0.5 mg/m^3	290.2 ± 12.3*	1.60 ± 0.14***
		1.5 mg/m^3	292.9 ± 7.4*	1.67 ± 0.09***
	D30	0 mg/m^3	318.8 ± 13.9	1.39 ± 0.14
		0.15 mg/m^3	327.3 ± 16.3	1.44 ± 0.09

	0.5 mg/m^3	314.8 ± 11.9	1.51 ± 0.10
	1.5 mg/m^3	322.1 ± 13.5	1.69 ± 0.11***
D180	0 mg/m^3	359.9 ± 21.9	1.56 ± 0.06
	0.15 mg/m^3	365.0 ± 28.0	1.54 ± 0.09
	0.5 mg/m^3	353.1 ± 20.9	1.60 ± 0.08
	1.5 mg/m^3	352.2 ± 29.6	1.68 ± 0.10*

Table S2: Respiratory parameters.

Respiratory parameters were measured on controls and exposed rats the week before exposure, the 1st and the 4th week of exposure, and after 1 week, 1 month and 6 months post-exposure.

	Time	Group	n	TV (ml)	tI (s)	tE (s)	PIf (ml/s)	PEf (ml/s)	f (breaths/min)	MV (ml)	tR (s)	EF50 (ml/s)	SRaw (cmH2Os)	dT (s)
P25	Before expo	Control	6	1.77 ± 0.18	0.16 ± 0.01	0.24 ± 0.03	15.5 ± 1.8	15.1 ± 2.6	164 ± 10	282 ± 28	0.18 ± 0.03	12.5 ± 3.7	1.16 ± 0.39	0.001 ± 0.000
		Exposed	6	1.87 ± 0.19	0.15 ± 0.01	0.20 ± 0.04	17.1 ± 1.0	15.9 ± 2.8	183 ± 14	334 ± 49	0.14 ± 0.03	13.8 ± 3.6	1.13 ± 0.29	0.001 ± 0.000
	1st week expo	Control	6	2.04 ± 0.29	0.19 ± 0.02	0.31 ± 0.04	15.4 ± 2.2	14.1 ± 3.1	135 ± 13	266 ± 41	0.23 ± 0.03	10.2 ± 2.8		
		Exposed	6	1.91 ± 0.25	0.18 ± 0.02	0.29 ± 0.04	14.6 ± 1.7	13.4 ± 1.2	141 ± 12	258 ± 23	0.21 ± 0.03	9.1 ± 0.7		
	4th week expo	Control	6	1.96 ± 0.33	0.18 ± 0.02	0.28 ± 0.05	15.8 ± 2.9	15.7 ± 4.2	154 ± 18	290 ± 68	0.20 ± 0.03	12.8 ± 4.5		
		Exposed	6	2.03 ± 0.18	0.19 ± 0.02	0.27 ± 0.03	15.5 ± 2.4	15.8 ± 1.8	147 ± 13	291 ± 42	0.20 ± 0.02	11.5 ± 2.1		
	1 week post-expo	Control	5	2.21 ± 0.28	0.16 ± 0.01	0.19 ± 0.04	18.9 ± 1.7	19.9 ± 2.3	188 ± 15	401 ± 37	0.13 ± 0.03	17.7 ± 2.8	1.30 ± 0.46	0.001 ± 0.000
		Exposed	6	2.36 ± 0.22	0.17 ± 0.01	0.19 ± 0.03	19.3 ± 1.5	19.0 ± 2.5	179 ± 14	414 ± 48	0.13 ± 0.02	17.2 ± 2.7	1.15 ± 0.23	0.001 ± 0.000
	1 month post-expo	Control	4	2.22 ± 0.34	0.18 ± 0.03	0.19 ± 0.04	17.9 ± 0.7	19.3 ± 1.0	179 ± 28	382 ± 8	0.13 ± 0.03	17.9 ± 1.7	1.64 ± 0.90	0.002 ± 0.001
		Exposed	5	2.50 ± 0.20	0.16 ± 0.01	0.19 ± 0.02	21.2 ± 2.0	20.2 ± 1.5	184 ± 13	446 ± 35	0.13 ± 0.02	18.5 ± 1.7	1.49 ± 0.30	0.002 ± 0.000
P90	Before expo	Control	6	2.09 ± 0.20	0.17 ± 0.02	0.19 ± 0.02	16.9 ± 2.2	17.4 ± 1.2	176 ± 16	359 ± 44	0.14 ± 0.02	15.3 ± 1.7	1.43 ± 0.45	0.002 ± 0.000
		Exposed	6	2.21 ± 0.07	0.17 ± 0.01	0.21 ± 0.03	18.0 ± 1.2	17.5 ± 1.4	173 ± 8	370 ± 20	0.15 ± 0.02	15.4 ± 2.2	1.33 ± 0.37	0.001 ± 0.000
	1st week expo	Control	6	2.09 ± 0.27	0.18 ± 0.01	0.29 ± 0.03	16.0 ± 2.3	17.5 ± 1.2	143 ± 4	290 ± 32	0.23 ± 0.02	11.6 ± 1.7		
		Exposed	6	1.88 ± 0.41	0.19 ± 0.03	0.27 ± 0.04	15.7 ± 3.6	13.0 ± 4.0	150 ± 18	271 ± 73	0.15 ± 0.03	11.0 ± 3.9		
	4th week expo	Control	6	2.03 ± 0.26	0.19 ± 0.02	0.32 ± 0.07	15.7 ± 2.1	17.1 ± 1.8	145 ± 20	279 ± 29	0.25 ± 0.05	11.9 ± 3.0		
		Exposed	5	1.75 ± 0.11	0.19 ± 0.02	0.31 ± 0.05	14.0 ± 0.9	11.9 ± 3.5	142 ± 12	239 ± 29	0.19 ± 0.03	9.7 ± 3.4		
	1 week post-expo	Control	6	2.04 ± 0.18	0.17 ± 0.01	0.20 ± 0.02	17.5 ± 1.1	18.0 ± 1.6	185 ± 18	363 ± 18	0.14 ± 0.02	15.5 ± 1.5	0.90 ± 0.24	0.001 ± 0.000
		Exposed	6	1.91 ± 0.13	0.15 ± 0.01	0.20 ± 0.02	18.5 ± 2.1	18.2 ± 1.1	204 ± 23	369 ± 24	0.15 ± 0.02	15.6 ± 1.3	1.20 ± 0.33	0.001 ± 0.000
	1 month post-expo	Control	5	1.88 ± 0.29	0.16 ± 0.01	0.18 ± 0.04	18.1 ± 3.7	18.9 ± 1.8	201 ± 10	370 ± 64	0.13 ± 0.03	17.1 ± 2.4	1.00 ± 0.25	0.001 ± 0.000
		Exposed	5	2.06 ± 0.12	0.16 ± 0.02	0.18 ± 0.02	17.4 ± 1.6	19.6 ± 2.1	190 ± 14	379 ± 16	0.12 ± 0.02	18.5 ± 2.3	1.28 ± 0.20	0.001 ± 0.000
	6 months post-expo	Control	3	1.82 ± 0.43	0.17 ± 0.02	0.25 ± 0.01	15.5 ± 1.7	16.3 ± 2.0	187 ± 43	302 ± 44	0.19 ± 0.02	13.3 ± 2.3	1.01 ± 0.14	0.001 ± 0.000
		Exposed	5	1.73 ± 0.13	0.16 ± 0.02	0.24 ± 0.05	16.3 ± 3.0	16.8 ± 3.0	187 ± 28	306 ± 71	0.18 ± 0.04	13.9 ± 3.7	1.06 ± 0.15	0.001 ± 0.000
Mitsui-7	Before expo	Control	6	2.14 ± 0.21	0.17 ± 0.02	0.23 ± 0.03	17.5 ± 2.0	16.3 ± 2.3	165 ± 15	324 ± 57	0.17 ± 0.03	13.6 ± 2.3	1.05 ± 0.41	0.001 ± 0.000
		Exposed	6	2.11 ± 0.23	0.17 ± 0.02	0.21 ± 0.01	16.3 ± 2.0	16.6 ± 1.4	166 ± 12	341 ± 32	0.15 ± 0.01	14.9 ± 1.7	1.49 ± 0.57	0.002 ± 0.001
	1st week expo	Control	6	2.13 ± 0.24	0.19 ± 0.01	0.32 ± 0.08	15.6 ± 2.7	16.8 ± 2.2	133 ± 16	275 ± 37	0.25 ± 0.07	10.8 ± 4.0		
		Exposed	6	1.91 ± 0.22	0.19 ± 0.02	0.32 ± 0.05	14.3 ± 1.1	15.7 ± 2.4	132 ± 16	244 ± 30	0.25 ± 0.04	9.3 ± 2.1		
	4th week expo	Control	6	2.05 ± 0.28	0.19 ± 0.02	0.32 ± 0.07	15.3 ± 1.6	15.7 ± 3.4	137 ± 19	269 ± 23	0.25 ± 0.05	10.8 ± 3.9		
		Exposed	6	1.94 ± 0.25	0.20 ± 0.02	0.36 ± 0.03	14.7 ± 1.7	13.6 ± 2.4	130 ± 11	237 ± 27	0.27 ± 0.03	8.2 ± 1.6		
	1 week post-expo	Control	6	2.31 ± 0.38	0.15 ± 0.02	0.18 ± 0.03	22.0 ± 3.0	20.6 ± 3.0	209 ± 24	454 ± 60	0.13 ± 0.02	18.7 ± 3.4	1.10 ± 0.18	0.001 ± 0.000
		Exposed	6	2.20 ± 0.30	0.15 ± 0.01	0.20 ± 0.04	20.0 ± 2.3	17.2 ± 2.9	189 ± 17	403 ± 58	0.14 ± 0.03	15.4 ± 3.4	1.53 ± 0.38	0.002 ± 0.000
	1 month post-expo	Control	4	2.14 ± 0.25	0.15 ± 0.02	0.19 ± 0.03	21.7 ± 2.4	20.0 ± 1.7	214 ± 28	429 ± 33	0.13 ± 0.03	17.6 ± 2.7	1.16 ± 0.33	0.001 ± 0.000
		Exposed	5	2.29 ± 0.29	0.16 ± 0.02	0.22 ± 0.03	21.4 ± 3.6	17.8 ± 2.3	191 ± 19	406 ± 62	0.16 ± 0.03	15.3 ± 2.6	1.12 ± 0.52	0.001 ± 0.001
	6 months post-expo	Control	4	1.61 ± 0.33	0.15 ± 0.03	0.22 ± 0.05	16.7 ± 2.8	15.5 ± 2.6	225 ± 57	306 ± 63	0.16 ± 0.04	12.7 ± 3.1	1.21 ± 0.33	0.001 ± 0.000
		Exposed	5	1.60 ± 0.23	0.15 ± 0.02	0.27 ± 0.05	16.3 ± 2.5	14.4 ± 2.0	194 ± 23	268 ± 31	0.21 ± 0.04	11.9 ± 2.8	1.29 ± 0.42	0.001 ± 0.000

Figures labelled in red: significantly different from the corresponding control ($p<0.05$).
TV: tidal volum, tI: inspiration time, tE: expiration time, PIf: peak inspiratory flow, PEf: peak expiratory flow, f: respiratory rate, MV: minute ventilation, tR: relaxation time, EF50: mid expiratory flow, SRaw: specific airway resistance, dT: time delay (between nasal and thoracic flows).

Supplementary method: respiratory parameters

The respiratory parameters of rats exposed to the highest concentration of P25, P90 and Mitsui-7 aerosols as well as those of their contemporary controls were monitored by plethysmography (from Electro-Medical Measurement Systems, Bordon, UK) the week prior the exposure, during the first and fourth week of exposure, then 1 week, 1 month and 6 months after the end of exposure. During inhalation, the animals were inside head-out plethysmographs (put directly on the inhalation towers) to measure the thoracic flow during exposure and to access the following parameters: tidal volume, inspiration and expiration times, peak inspiratory and expiratory flows, breathing frequency, minute volume, relaxation time and mid-tidal expiratory flow. In addition, double-chamber plethysmographs were used outside the exposure periods to measure nasal and thoracic flows allowing evaluation of time delay (between nasal and thoracic flows) and the specific airway resistance. Parameters were measured from around 30 min after the animals were placed in the tubes and for at least one hour. Measurement of respiratory parameters 6 months after exposure to P25 could not be undertaken because the size of the plethysmography tubes (PLY 222) could not accommodate the animals. Larger tubes (PLY 222 L) were acquired to allow these measurements on the P90 and Misui-7 animals. All statistical analyses were performed using Statgraphics Centurion XVIII Software (Version 18.1.06) (Statgraphics Technologies, Inc., The Plains, Virginia). Data are expressed as the mean ± standard deviation. For each exposure campaign (P25, P90 and Mitsui-7), two-factor (group and time) analyses of variance were used to analyze each respiratory parameter. When the interaction was significant, the effect of the group was tested for each timepoint, applying a Bonferroni correction. The threshold for statistical significance was set at 5%.

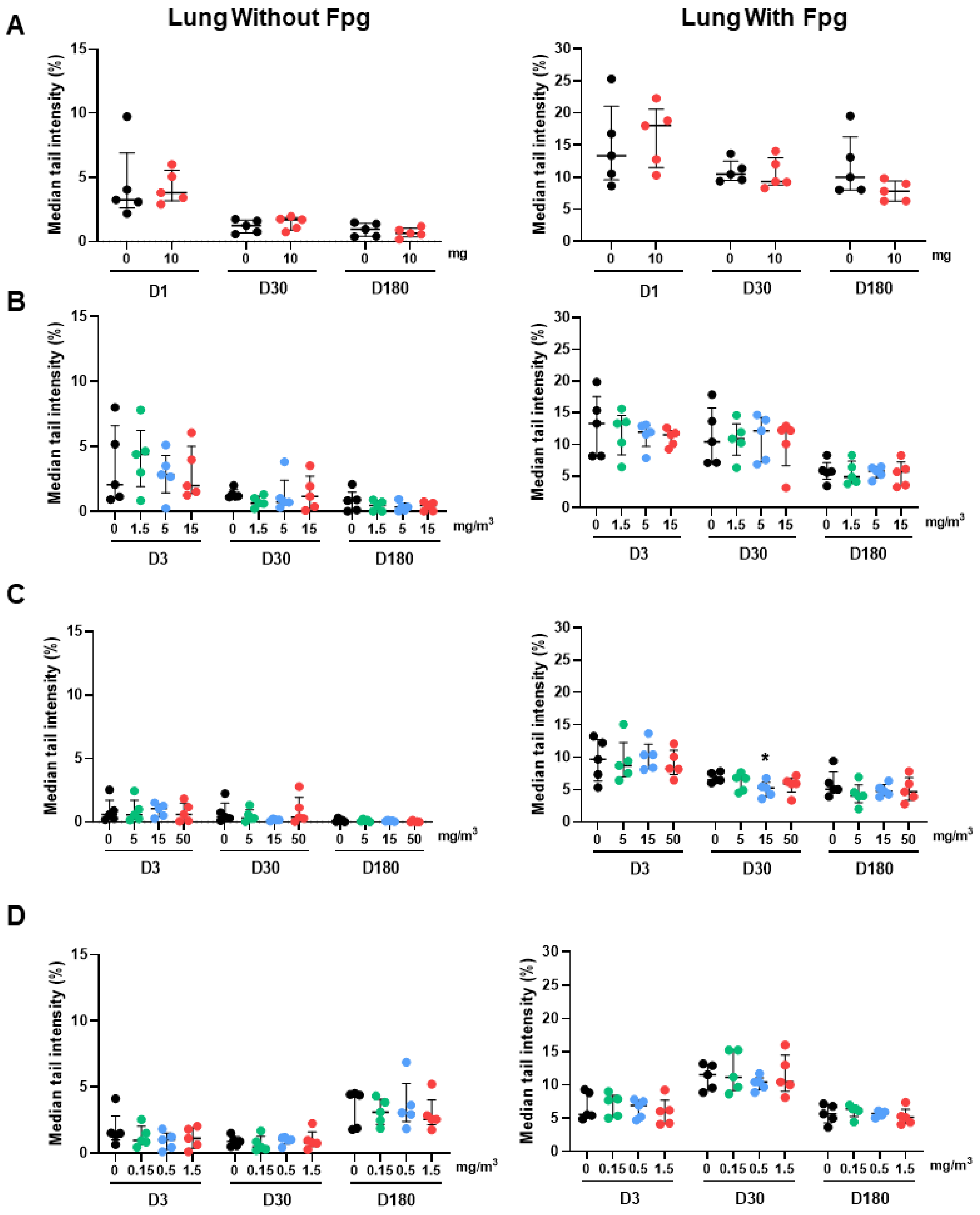
A
Lung Without Fpg
Lung With Fpg
Median tail intensity (%)
mg
D1
D30
D180
B
mg/m3
D3
D30
D180
C
*
D
0.15
0.5
1.5

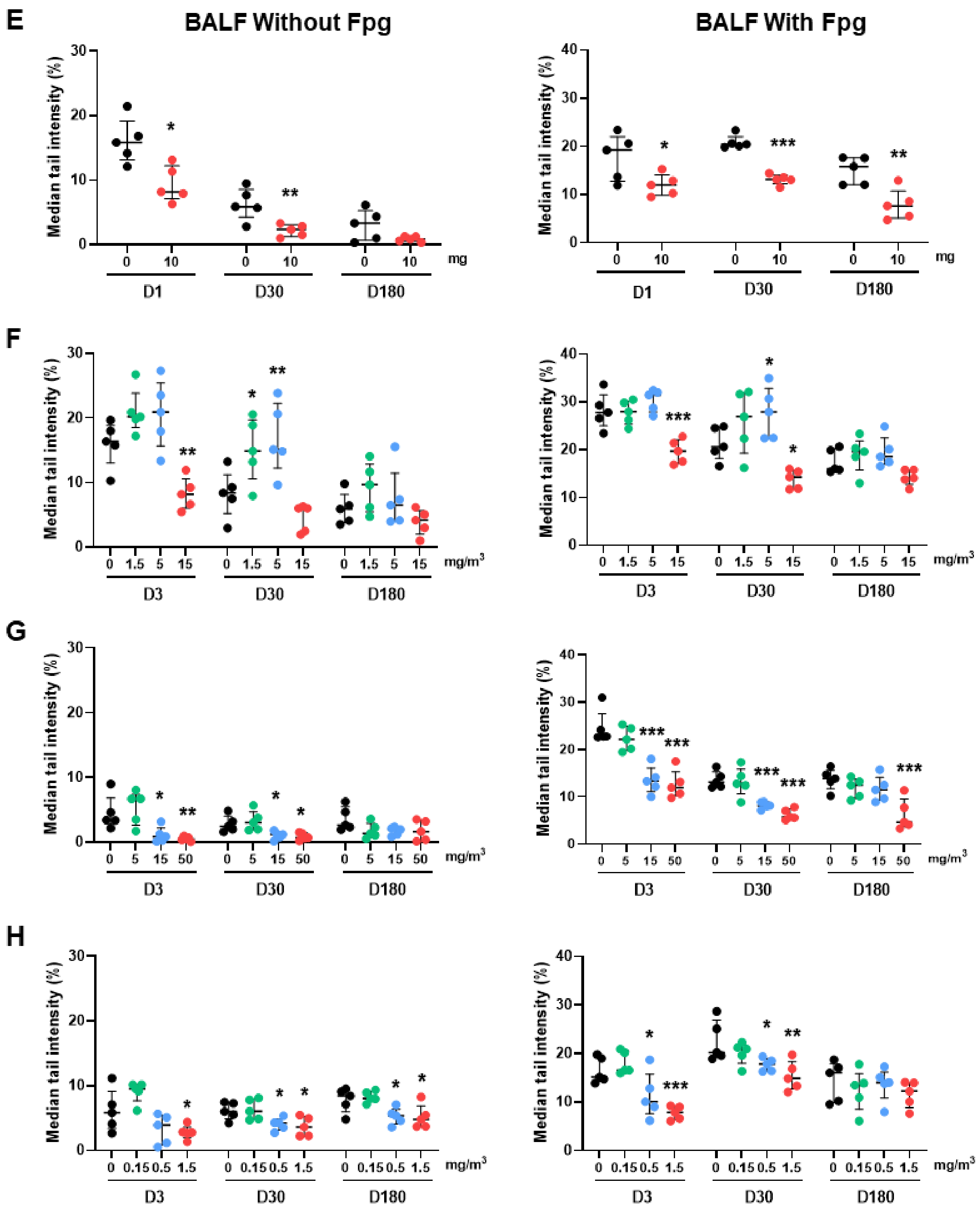

Figure S1: DNA damage in lung tissue and BALF.

The comet assay was assessed on lung (A – D) and BALF (E – H) with and without FPG at the times indicated after the end of exposure to 10 mg/rat of DQ-12 (A and E), to 1.5, 5 or 15 mg/m^3 of P25 (B and F), to 5, 15 or 50 mg/m^3 of P90 (C and G) and to 0.15, 0.5 or 1.5 mg/m^3 of Mitsui-7 (D and H). The results are presented as median tail intensity (%). Data are expressed as median [Q1; Q3]. * $p<0.05$, ** $p<0.01$, *** $p<0.001$ significantly different from the control (n = 5 except for Mitsui-7 medium dose D180 n=4).

Supplementary method: comet assay

After BAL, the left lung was cut into small pieces and transferred into the C-Tube containing Collagenase type IA (1 mg/mL in Krebs buffer, Sigma Aldrich #C9891). After the first dissociation program (Miltenyi Biotec Gentle Max dissociator), the C-Tube was incubated for 20 min at 37°C. After an additional program of dissociation, the suspension was filtered successively through 100 and 70 µm strainers. The cells were centrifuged for 5 min at 400*g* and resuspended in Krebs buffer. DNA strand breaks were determined on BAL fluid and lung cells (n = 5) as described previously (1). Cells were mixed with 1% low-gelling agarose (Sigma Aldrich #A9414) at a final concentration of 100,000 cells/mL and poured onto microscope glass slides pre-coated with 1% routine agarose (Sigma Aldrich #A9539). The slides were immersed overnight at 4°C in the ice-cold lysis buffer (2.5 mM NaCl; 100 mM Na_2EDTA; 10 mM Tris base; 10% DMSO and 1% Triton; pH 10). For each animal two slides were prepared: one for the regular single gel electrophoresis assay and one for the formamidopyrimidine DNA glycosylase (FPG)-modified comet assay. For the FPG-modified comet assay, following lysis, slides were washed three times at 4°C with the FPG incubation buffer (40 mM Hepes; 0.1 M KCl; 0.5 mM EDTA; 0.2 mg/mL bovine serum albumin; pH 8) followed by incubation for 30 min at 37°C with 5 U/mL of FPG (Sigma Aldrich #F3174). For the regular comet assay, slides were incubated with only the FPG incubation buffer for 30 min at 37°C. The slides were then immersed for 20 min in the ice-cold alkaline electrophoresis buffer (300 mM NaOH; 1 mM Na_2EDTA; pH > 13) and subjected to electrophoresis for 40 min at 0.9 V/cm. At the end of electrophoresis, the slides were immersed in the neutralization buffer (0.4 M Tris base; pH 7.5) for 15 min at 4°C. The slides were washed with cold ultrapure water, dehydrated in cold 96% ethanol, dried at 56°C and stored in the dark at room temperature. For fluorescent microscopy analysis, the slides were rehydrated with cold ultrapure water and stained with propidium iodide 2.5 µg/mL (Sigma Aldrich #70335). For each sample, 200 cells were analyzed using the Comet Assay IV software (Perceptive Instruments,Suffolk, UK) and the percentage of DNA in the tail of each comet was measured. The positive controls were BAL and lungs cells from rats exposed by gavage to methyl methanesulfonate (Sigma Aldrich #129925) at a final concentration of 12.5 mg/kg b.w. 3h before tissue collection.

Reference :

1. Guichard Y, Maire MA, Sebillaud S, Fontana C, Langlais C, Micillino JC, et al. Genotoxicity of Synthetic Amorphous Silica Nanoparticles in Rats Following Short-Term Exposure, Part 2: Intratracheal Instillation and Intravenous Injection. Environmental and Molecular Mutagenesis. 2015;56(2):228-44.

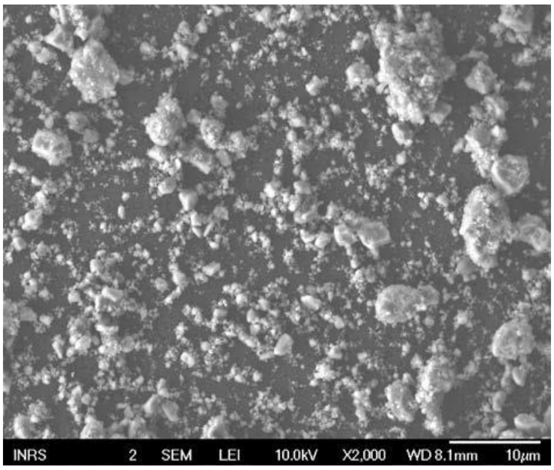

Figure S2: Scanning electron microscopy of DQ-12 particles.
Presence of sub-micron particles.

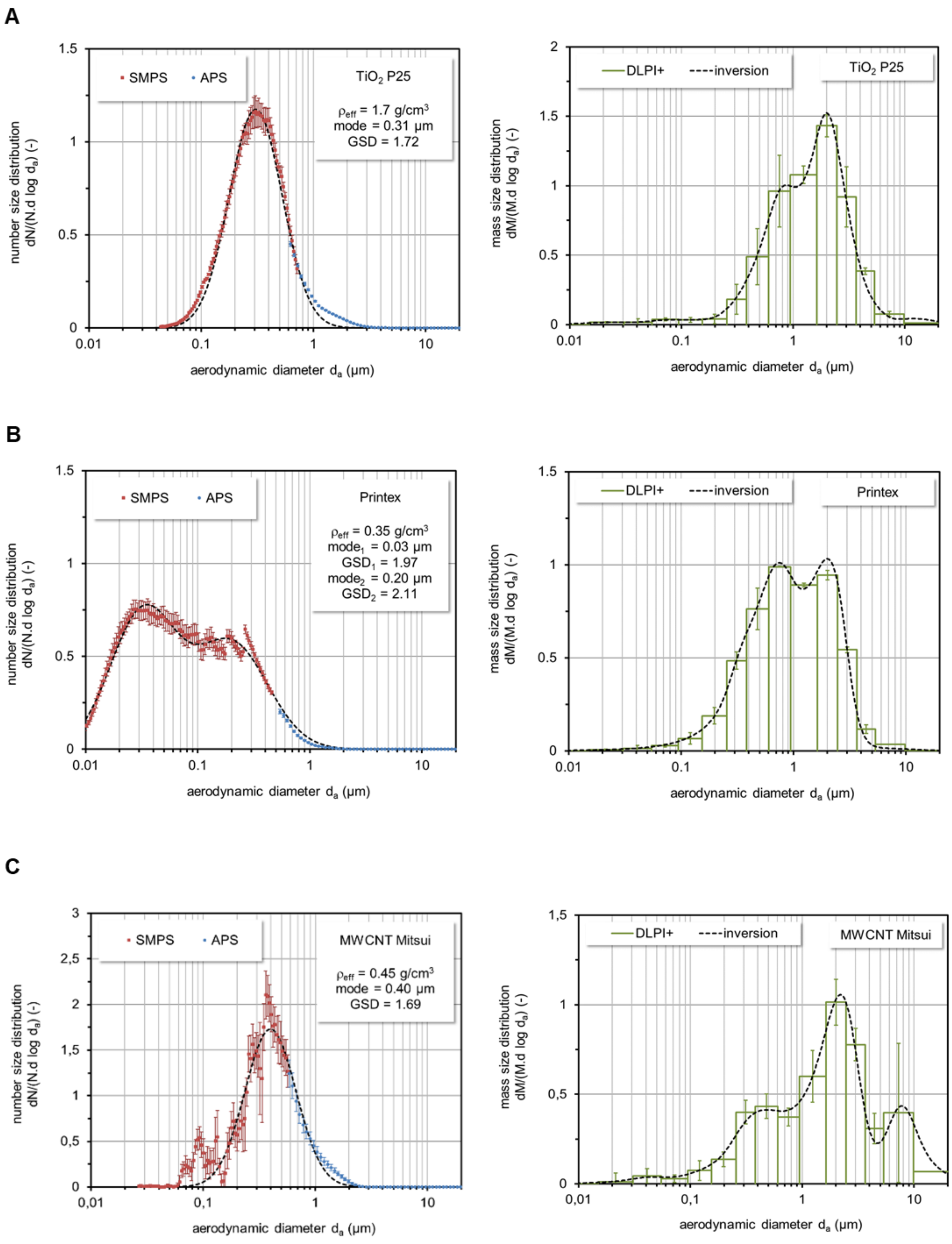

Figure S3: Number and mass size distribution of the aerosols.
The number (left panels) and mass (right panels) size distribution of P25 (A), P90 (B) and Mitsui-7 (C) aerosol are presented.

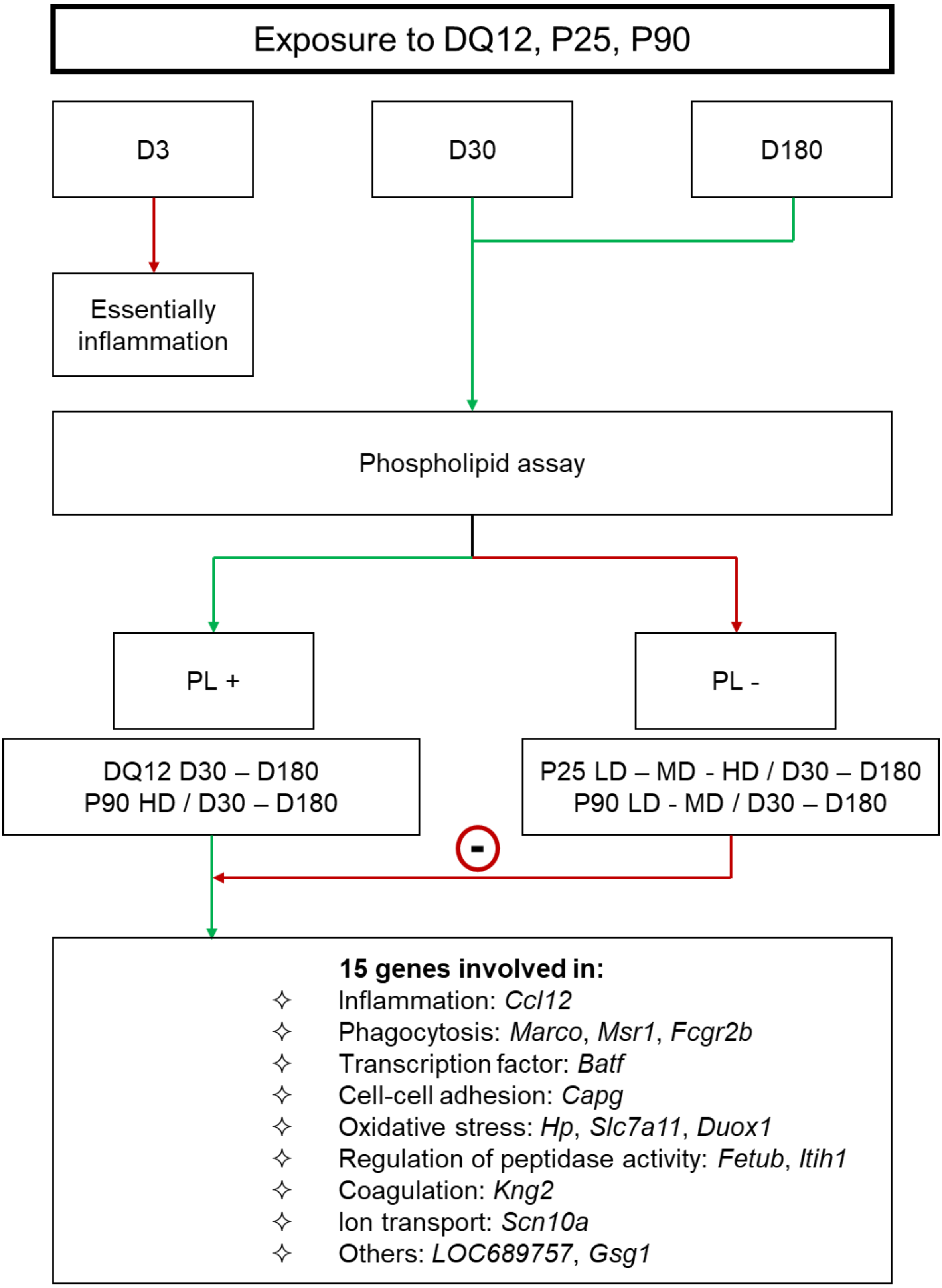

Figure S4: Filtration strategy of differentially expressed genes associated with phospholipid accumulation.

Following exposure to DQ-12, low dose (LD), medium dose (MD) or high dose (HD) of P25 or P90, transcriptome was analyzed on lung rats 3 (D3), 30 (D30) and 180 (D180) days post-exposure. Phospholipid assay was used to distinguish samples with a statistically significant

increase of BALF phospholipids as compared to control groups (PL+) or not (PL-). DEGs: differentially expressed genes.
